# Toxoplasmosis in Transplant Recipients, Europe, 2010–2014

**DOI:** 10.3201/eid2408.180045

**Published:** 2018-08

**Authors:** Florence Robert-Gangneux, Valeria Meroni, Damien Dupont, Françoise Botterel, José M. Aguado Garcia, Marie-Pierre Brenier-Pinchart, Isabelle Accoceberry, Hamdi Akan, Isabella Abbate, Katia Boggian, Fabrizio Bruschi, Jordi Carratalà, Miruna David, Lubos Drgona, Olgica Djurković-Djaković, Maria Carmen Farinas, Francesca Genco, Effrossyni Gkrania-Klotsas, Andreas H. Groll, Edward Guy, Cédric Hirzel, Nina Khanna, Özgür Kurt, Lia Monica Junie, Tiziana Lazzarotto, Oscar Len, Nicolas J. Mueller, Patricia Munoz, Zoi Dorothea Pana, Emmanuel Roilides, Tijana Stajner, Christian van Delden, Isabelle Villena, Hervé Pelloux, Oriol Manuel

**Affiliations:** Université Rennes, Rennes, France (F. Robert-Gangneux);; Centre Hospitalier Universitaire de Rennes, Rennes (F. Robert-Gangneux);; European Society of Clinical Microbiology and Infectious Diseases, Basel, Switzerland (V. Meroni, J.M. Aguado Garcia, F. Bruschi, J. Carratalà, M. David, L. Drgona, E. Gkrania-Klotsas, A.H. Groll, Ö. Kurt, L.M. Junie, O. Len, P. Munoz, Z.D. Pana, E. Roilides, I. Villena, H. Pelloux, O. Manuel);; University of Pavia, Pavia, Italy (V. Meroni, F. Genco);; Hôpital de la Croix-Rousse, Lyon, France (D. Dupont);; Université Claude Bernard Lyon, Lyon (D. Dupont);; Centre Hospitalier Universitaire Henri Mondor, Creteil, France (F. Botterel);; University Hospital 12 de Octubre Universidad Complutense, Madrid, Spain (J.M. Aguado Garcia, H. Akan);; Centre Hospitalier Universitaire Grenoble-Alpes, Grenoble, France (M.-P. Brenier-Pinchart);; Centre Hospitalier Universitaire de Bordeaux, Bordeaux, France (I. Accoceberry);; Ankara University Cebeci Hospital, Ankara, Turkey (H. Akan);; Lazzaro Spallanzani National Institute for Infectious Diseases, Rome, Italy (I. Abbate);; St. Gallen Cantonal Hospital, St. Gallen, Switzerland (K. Boggian);; Pisa University, Pisa, Italy (F. Bruschi);; Bellvitge University Hospital-IDIBELL University of Barcelona, Barcelona, Spain (J. Carratalà);; Queen Elizabeth Hospital, Birmingham, UK (M. David);; Comenius University, Bratislava, Slovakia (L. Drgona); National Cancer Institute, Bratislava (L. Drgona);; University of Belgrade, Belgrade, Serbia (O. Djurković-Djaković, T. Stajner);; Hospital Universitario Marques de Valdecilla, Santander, Spain (M.C. Farinas);; University of Cantabria, Santander (M.C. Farinas);; Addenbrooke’s Hospital, Cambridge, UK (E. Gkrania-Klotsas);; University Children’s Hospital Münster, Münster, Germany (A.H. Groll);; Singleton Hospital, Swansea, Wales, UK (E. Guy);; Bern University Hospital, Bern, Switzerland (C. Hirzel);; University of Bern, Bern (C. Hirzel); Universitätsspital, Basel (N. Khanna);; Acibadem Mehmet Ali Aydinlar, Istanbul, Turkey (Ö. Kurt);; University of Medicine and Pharmacy Iuliu Hatieganu, Cluj Napoca, Romania (L.M. Junie);; St. Orsola-Malpighi General Hospital, Bologna, Italy (T. Lazzarotto);; University of Bologna, Bologna (T. Lazzarotto);; Hospital Universitari Vall d’Hebron, Barcelona (O. Len);; Zurich University Hospital, Zurich, Switzerland (N.J. Mueller);; University of Zurich, Zurich (N.J. Mueller);; Hospital General Gregorio Maranon, Madrid, Spain (P. Munoz);; Hippokration Hospital, Aristotle University of Thessaloniki, Thessaloniki, Greece (Z.D. Pana, E. Roilides);; University Hospitals Geneva, Geneva, Switzerland (C. van Delden);; Centre Hospitalier Universitaire de Reims, Reims, France (I. Villena);; University Hospital, Lausanne, Switzerland (O. Manuel);; University of Lausanne, Lausanne (O. Manuel)

**Keywords:** toxoplasma infection, transplantation, transplant recipient, cerebral toxoplasmosis, hematopoietic stem cell transplant, chemoprophylaxis, cotrimoxazole, parasites, Europe, Toxoplasma

## Abstract

Transplantation activity is increasing, leading to a growing number of patients at risk for toxoplasmosis. We reviewed toxoplasmosis prevention practices, prevalence, and outcomes for hematopoietic stem cell transplant (HSCT) and solid organ transplant (SOT; heart, kidney, or liver) patients in Europe. We collected electronic data on the transplant population and prevention guidelines/regulations and clinical data on toxoplasmosis cases diagnosed during 2010–2014. Serologic pretransplant screening of allo-hematopoietic stem cell donors was performed in 80% of countries, screening of organ donors in 100%. SOT recipients were systematically screened in 6 countries. Targeted anti-*Toxoplasma* chemoprophylaxis was heterogeneous. A total of 87 toxoplasmosis cases were recorded (58 allo-HSCTs, 29 SOTs). The 6-month survival rate was lower among *Toxoplasma*-seropositive recipients and among allo-hematopoietic stem cell and liver recipients. Chemoprophylaxis improved outcomes for SOT recipients. Toxoplasmosis remains associated with high mortality rates among transplant recipients. Guidelines are urgently needed to standardize prophylactic regimens and optimize patient management.

Toxoplasmosis is a zoonosis that infects humans and other warm-blooded animals worldwide; prevalence and clinical severity vary by geographic area (*1*). After primary infection, the parasite persists lifelong within dormant tissue cysts. Transmission to humans mainly occurs by ingestion of food or water contaminated with oocysts from feces of infected felids or undercooked meat containing cysts (*2*). Although largely asymptomatic in adults, toxoplasmosis is a life-threatening opportunistic infection in immunocompromised patients of all ages. Similar to *Pneumocystis* pneumonia, toxoplasmosis has become more frequently diagnosed for patients receiving immunosuppressive therapy than for patients with HIV infection (*3*,*4*). The growing number of grafts makes transplant patients a population at increasing risk. In transplant recipients (solid organ transplant [SOT] or hematopoietic stem cell transplant [HSCT]), disease can result from reactivation of past latent infection or from primary infection acquired through contaminated food or through a transplanted organ containing latent cysts (*5*). In contrast to incidence among HIV-infected patients, the incidence of toxoplasmosis among transplant recipients is poorly documented; published studies reporting patient series are scarce (*6*–*8*), and the literature consists mostly of case reports (*2*,*9*–*12*). 

The risk for reactivation of chronic infection varies according to the immunosuppressive protocol and therefore according to the type of graft (*13*); risk is highest for seropositive allo-HSCT recipients receiving a seronegative graft. Among SOT recipients, the risk of a seronegative recipient (R–) acquiring infection from a seropositive donor (D+) organ (D+/R–) depends on the organ type; risk is highest for heart transplant recipients. Prevention measures rely on pretransplant serologic screening of donor, recipient, or both and on chemoprophylaxis; however, guidelines and regulations differ largely among countries. Regarding chemoprophylaxis, a multicenter study in France revealed variable practices in terms of regimen and duration of treatment (*4*). Some experts have proposed a tight clinical and molecular follow-up protocol for HSCT patients, aiming at early diagnosis of *Toxoplasma* reactivation to improve survival rates (*14*–*16*), but the cost:benefit ratio of this strategy is still under debate. We reviewed prevention practices implemented in European countries and evaluated the burden of toxoplasmosis among HSCT and SOT recipients.

## Methods

### Participating Centers

We recruited applicants through 2 study groups of the European Society of Clinical Microbiology and Infectious Diseases (ESCMID; the European Study Group on Clinical Parasitology and the European Study Group on Immunocompromised Hosts) and through the Spanish Transplantation Infection Study Group, the Italian Society of Clinical Microbiology Infections and Transplant Working Group, and the Swiss Transplant Cohort Study. For each country, a local coordinator was identified and was in charge of contacting investigators from transplantation or infectious diseases units from representative centers.

### Data Collection

Participants were invited to answer a detailed questionnaire adapted to the type of graft and designed to collect the following information: *Toxoplasma* seroprevalence in the country (documented by articles or recent surveys); implementation of a case reporting system for toxoplasmosis in transplant recipients; annual number of transplant procedures for each organ type in the participating center and in the whole country; pretransplant serologic screening policy for recipients and donors; implementation of recipient monitoring after transplantation and methods used (PCR, serology); and chemoprophylaxis regimen and duration according to organ type (if cotrimoxazole was given primarily for preventing *Pneumocystis* pneumonia, this use was recorded) and according to the recipient serologic results (primary or secondary prophylaxis, whether chemoprophylaxis was given to seronegative recipients, seropositive recipients, or both). When official national guidelines were lacking, to obtain representative data, we collected information about local practices in several transplant centers whenever possible. 

As a second step, we sent an electronic case reporting form to all voluntary participating centers, which retrospectively recorded the number of cases of toxoplasmosis diagnosed per center over a 5-year period (2010–2014). The form collected the following information: patient age and sex; date of transplantation and type of graft; *Toxoplasma* serologic status of recipient and donor; date of toxoplasmosis diagnosis; site of infection (cerebral, ocular, disseminated); tools contributing to diagnosis (serology, molecular diagnosis, pathology, direct examination, imaging); chemoprophylaxis type, date of initiation, and duration; patient survival at 2 and 6 months; and date of death, if applicable. From each center and for each organ type, we also collected the mean patient age and the mean percentage of the whole transplant patient population surviving at 2 months and at 6 months. The number of cases and clinical data were retrieved from hospital medical or laboratory databases or from local or national databases, if existing. Participants were invited to send only aggregated data generated automatically by the database. The study was approved by the Ethics Committee of the University Hospital of Rennes, France (approval no. 15.12).

### Statistical Analyses

Descriptive statistics are expressed as frequency (percentage) or mean ± SE. Comparison of qualitative data between groups was based on exact χ^2^ tests for equal proportions or Fisher exact tests if single table values were <5; quantitative data were compared by using analysis of variance or *t*-test (nonparametric test). We computed data by using SAS software version 9.4 (SAS Institute, Cary, NC, USA).

## Results

### Participating Centers, Transplantation Activity, and Case Notification 

Overall, 46 centers from 11 countries (1–10 centers/country) participated in the survey; countries represented were France, Germany, Greece, Italy, Romania, Serbia, Slovakia, Spain, Switzerland, Turkey, and the United Kingdom (Technical Appendix). Responses indicated that 5 countries (Switzerland, Slovakia, Turkey, Greece and the United Kingdom) report toxoplasmosis cases in a national database.

During 2010–2014, the mean annual number (range) of allo-HSCT procedures reported per country was 1,016 (13–1,900) and of auto-HSCT was 1,524 (14–3,078) (online Technical Appendix). Regarding SOT recipients, the mean annual number (range) of transplantations was 155 (10–420) for heart, 1,286 (55–3,074) for kidney, and 622 (35–1,241) for liver. The cumulative annual transplant activity among the responding centers reached a total of 1,089 allo-HSCT and 1,168 auto-HSCT (26 centers) and, for SOT, 394 heart (26 centers), 2,566 kidney (35 centers), and 1,455 liver (26 centers) transplants.

### Pretransplant Serologic Screening for Toxoplasmosis

Although serologic screening of HSCT donors is not mandatory, all responding countries reported that they were performing this screening. Screening of allo-HSCT recipients was performed in all countries except Slovakia (mandatory in 4 countries), whereas screening of auto-HSCT recipients was performed regularly (4 countries), inconstantly (5 countries), or not at all (1 country). Overall, of 26 responding centers, 24 centers screened allo-HSCT and 17 screened auto-HSCT recipients for *Toxoplasma* antibodies before transplantation.

Serologic screening of solid organ donors (heart, kidney, or liver) was performed in all countries, although screening was reportedly mandatory in only 7 countries (France, Greece, Italy, Romania, Slovakia, Switzerland, and Turkey). At most centers, SOT recipients were screened (24/26 liver, 31/35 kidney, and 25/26 heart).

### Anti-*Toxoplasma* Chemoprophylaxis Practices and Follow-up

Virtually all allo-HSCT recipients received cotrimoxazole chemoprophylaxis, whether primarily targeting *Pneumocystis* or *Toxoplasma*. At the 24 responding centers, cotrimoxazole was usually prescribed for >6 months despite the lack of official guidelines at 11 (46%) centers. The preferred regimen at 60% of centers was 960 mg 3 times a week but ranged from 480 mg 2 times a week to 1,920 mg 3 times a week. Auto-HSCT patients at 73% of centers received cotrimoxazole, administered mostly for 3 or 6 months. Serologic follow-up was reported by 2 allo-HSCT centers and PCR-based follow-up by 4.

For heart transplant recipients, 24 (92%) of 26 centers stated that they gave cotrimoxazole prophylaxis (3 months to lifelong), and 10 (43%) of 23 centers implemented serologic follow-up for *Toxoplasma* 2 and 4 times per year, particularly in cases of serologic mismatch (D+/R–). The most frequently prescribed regimen was 960 mg of cotrimoxazole 3 times a week or 480 mg daily. Although anti-*Pneumocystis* prophylaxis was implemented at 29 (83%) of 35 kidney and 17 (65%) of 26 liver transplant centers for 3–12 months, specific recommendations regarding toxoplasmosis chemoprophylaxis in this population were reported by only 4 countries (France, Greece, Spain, Turkey). The most frequently used regimen was cotrimoxazole at 480 mg daily (50% of kidney and 40% of liver transplant centers). Serologic monitoring of D+/R– patients was reportedly performed at 6 kidney and 5 liver transplant centers.

### Incidence and Clinical Presentation of Toxoplasmosis

Overall, during the 5-year study period, 87 cases of *Toxoplasma* infection in transplant patients (58 HSCT, 29 SOT) were reported from 15 centers in 8 countries (online Technical Appendix). Severe manifestations (cerebral toxoplasmosis, disseminated toxoplasmosis, pulmonary toxoplasmosis) were more frequently observed (42 [48%] patients) than were mild manifestations (ocular toxoplasmosis, fever; 14 [16%] patients). A total of 31 (36%) patients had no apparent clinical signs. Asymptomatic episodes occurred mainly among HSCT recipients (81%) and were diagnosed mostly on the basis of a positive PCR (84%). Symptomatic HSCT recipients most often had disseminated (10/33, 30%) or cerebral (11/33, 33%) toxoplasmosis; these cases accounted for 60% of all cases of disseminated and 85% of cerebral toxoplasmosis (Table 1).

**Table 1:** Characteristics of 87 transplant patients with toxoplasmosis, according to clinical presentation, Europe, 2010–2014*

Variables	Clinical type						p value
Cerebral	Ocular	Disseminated	Pulmonary	Fever alone	No signs
No. (%) patients	13 (15)	4 (5)	19 (22)	10 (11)	10 (11)	31 (36)	
Patient age, y, mean ± SE	37.0 ± 7.7	60.7 ± 0.8	47.8 ± 5.6	53.1 ± 4.8	35.5 ±4.4	46.4 ± 4.2	<0.0001
Time graft/diagnosis, wk, mean ± SE	123 ± 151	313 ± 175	163 ± 124	19 ± 11	73 ± 43	99 ± 51	<0.05
Diagnosis by, no. (%)							
PCR	13 (100)	3 (75)	17 (89)	9 (90)	9 (90)	26 (84)	<0.001
Serology	3 (23)	3 (75)	9 (47)	2 (20)	5 (50)	5 (16)	0.2278
Imaging	12 (92)	3 (75)	8 (42)	7 (70)	0	2 (6)	<0.01
Microscopy	1 (8)	0	6 (32)	1 (10)	0	0	<0.01
Graft type, no. (%)							<0.05
Liver, n = 8	1 (8)	1 (25)	3 (16)	2 (20)	0	1 (3)	
Kidney, n = 9	1 (8)	1 (25)	1 (5)	2 (20)	3 (30)	1 (3)†	
Heart, n = 12	0	1 (25)	5 (26)	0	2 (20)	4 (13)‡	
Allo-HSC, n = 58	11 (85)	1 (25)	10 (53)	6 (60)	5 (50)	25 (81)§	
No. with mismatch, n = 11	0	1 (25)	5 (26)	1 (10)	4 (40)	0	<0.05
Survival, no. (%)							
2 mo	5 (38)	4 (100)	13 (68)	5 (50)	10 (100)	24 (77)	<0.0001
6 mo	2 (15)	4 (100)	10 (53)	5 (50)	7 (70)	18 (58)	<0.001

*HSC, hematopoietic stem cell. †This patient was receiving chemoprophylaxis. ‡1 patient was receiving chemoprophylaxis.  §11 patients were receiving chemoprophylaxis.

For the 87 reported cases, PCR was the most helpful diagnostic tool (77 [89%] cases), followed by imaging (32 [37%] cases) and serology (28 [32%] cases) (Table 1). PCR was reportedly positive for 100% of patients with cerebral and 90% with pulmonary toxoplasmosis (Table 1).

Pretransplantation *Toxoplasma* serologic test results for donor and recipient were available for 70 of the 87 patients (46 HSCT and 24 SOT). Toxoplasmosis occurred in the main groups at risk: in 35 (76%) of 46 D–/R+ HSCT recipients and 11 (46%) of 24 D+/R– SOT recipients (Table 2). Overall, 35 patients (18 HSCT and 17 SOT recipients) received chemoprophylaxis (Table 3). Only 4 (36%) of 11 D+/R– SOT recipients received chemoprophylaxis, but for all of them toxoplasmosis occurred after discontinuation of prophylaxis (data not shown). Overall, toxoplasmosis was diagnosed after the end of prophylaxis for 17 recipients (9 HSCT and 8 SOT). For 9 HSCT and 5 SOT recipients, toxoplasmosis occurred during chemoprophylaxis (Table 3). Of these, 13 (93%) were asymptomatic: 1 kidney, 1 heart, and 11 HSC transplant recipients (Table 1). The proportion of mismatched cases (D+/R–) did not differ according to organ type (Table 4).

**Table 2:** Characteristics of transplant donors and recipients at transplantation, according to *Toxoplasma* serologic status, Europe, 2010–2014*

Serologic status of donor/recipient†	Prophylaxis, no. (%)	Graft type, no.				Survived 6 mo, no. (%)	Wks between diagnosis and graft, mean ± SE
Liver	Kidney	Heart	HSC
Positive/positive, n = 9‡	5 (56)	2	0	1	6	3 (33)	21 ± 9
Positive/negative, n = 11§	4 (36)	3	4	4	0	9 (82)	309 ± 275
Negative/positive, n = 36¶	12 (33)	0	0	1	35	12 (33)	15 ± 3
Negative/negative, n = 14	9 (64)	2	2	5	5	11 (79)	123 ± 31
p value	0.1975	NA	NA	NA	NA	<0.01 (0.0029)	<0.05

*HSC, hematopoietic stem cell; NA, not applicable. †Missing data for 17 patients.  ‡2 liver transplant recipients died.  §Group in which solid organ transplant patients are most at risk for toxoplasmosis.  ¶Group in which HSC transplant patients are most at risk for toxoplasmosis.

**Table 3:** Toxoplasmosis occurrence and outcomes for HSCT and SOT patients, according to prophylaxis, Europe, 2010–2014*

Characteristic	HSCT, no. (%), n = 58†	SOT, no. (%), n = 29‡	p value
Seropositive before transplantation	41/46 (89)§	4/24 (17)¶	<0.0001
Diagnosis during chemoprophylaxis	9/50 (18)	5/28 (18)	NS
Diagnosis after chemoprophylaxis	9/50 (18)	8/28 (29)	NS
2-mo survival rate			
With prophylaxis	13/18 (72)	17/17 (100)	<0.05
Without prophylaxis	18/32 (56)	9/11 (82)	0.1657
6-mo survival rate			
With prophylaxis	9/18 (50)	17/17 (100)	0.01
Without prophylaxis	9/32 (28)	7/11 (64)#	0.0679

*HSCT, hematopoietic stem cell transplant; NS, not significant; SOT, solid organ transplant. †Prophylaxiis data missing for 8 patients. ‡Prophylaxis data missing for 1 patient; incomplete information regarding dates of onset and/or stop of cotrimoxazole for 4 patients. §Serology data missing data for 4 patients.  ¶Serology data missing data for 4 patients.  #p<0.05 between SOT with or without chemoprophylaxis.

**Table 4:** Characteristics of transplant patients with toxoplasmosis, according to graft type and comparison to overall graft population, Europe, 2010–2014*

Characteristics	Allo-HSC			Kidney			Liver			Heart		p value
	Case-patients	All TP		Case-patients	All TP		Case-patients	All TP		Case-patients	All TP	
Patients, no.	58	4,108		9	6,507		8	2,983		12	998	NA
Age, y, mean ± SE	46.8 ± 5.3	50.7		44.6 ± 5.9	50.9		55.1 ± 1.5	51.6		44.4 ± 6.4	47.5	NA
Female sex, %	43	38		44	63		38	30		17	22	NA
Male sex, %	57	62		56	37		62	70		83	78	NA
Mean time diagnosis/graft, wk, mean ± SE	20.6 ± 4.6	NA		198 ± 68	ND		152 ± 144	ND		441 ± 155	ND	<0.0001
Mean time diagnosis/death, d, mean ± SE	47 ± 18	NA		33	ND		38 ± 17	ND		NA	ND	0.8595
Mismatched serologic results (D+/R–), no. (%)	0	NA		4 (33)	NA		3 (38)	NA		4 (33)	NA	0.8923
2-mo survival, no. (%)	36 (62)	ND		8 (89)	ND		5 (63)	ND		12 (100)	ND	<0.05
Deep site involvement	12 (43)	ND		3 (60)	ND		4 (57)	ND		6 (100)	ND	0.0513
Fever only	5 (100)	ND		3 (100)	ND		0	ND		2 (100)	ND	1
No clinical signs	18 (72)			1 (100)			1 (100)			4 (100)		0.1407
6-mo survival, %†	38	84‡		89	72		50	75§		100	60	<0.0001
Deep site involvement	25	NA		60	NA		28	NA		100	NA	<0.01
Fever only	40	NA		100	NA		0	NA		100	NA	0.2083

*D+, donor positive; NA, not applicable; ND, not determined; NS, not significant; R–, recipient negative; TP, transplant patients. †The survival rate for the general TP population was calculated at time similar to the mean time of diagnosis of toxoplasmosis after graft in case-patients. ‡p<0.01 compared with case-patients.  §p<0.05 compared with case-patients.

The mean time between transplantation and toxoplasmosis diagnosis was shorter among patients with pulmonary toxoplasmosis (p<0.05) (Table 1) than among patients with other types of disease manifestation. For seropositive recipients, the mean time to toxoplasmosis onset was short (<4 months after transplantation) compared with that for seronegative recipients (>4 years) (Table 2). Furthermore, the time to disease onset after transplantation was shorter among HSCT patients than SOT recipients (p<0.0001) (Table 4). The incidence of toxoplasmosis differed among the responding countries but seemed to not be linked to the seroprevalence in the country (online Technical Appendix).

### Risk Factors for Death 

Survival rates differed significantly between HSCT and SOT recipients (p<0.001) (Table 5). The 2-month survival rate was significantly poorer for patients with cerebral (38%) or pulmonary (50%) toxoplasmosis (p<0.001) (Table 1). Survival rates were also poorer for seropositive patients (p<0.05 at 2 months and p<0.001 at 6 months) (Table 5), mainly consisting of HSCT patients (Table 2). Of note, the percentage of asymptomatic patients who survived 6 months (58%) was similar to that of patients with pulmonary (50%) or disseminated (53%) toxoplasmosis (Table 1). A lower percentage of HSCT and liver transplant recipients survived at 2 and 6 months after diagnosis; deep site–associated toxoplasmosis was diagnosed for only half of them (Table 4). The survival rates for HSCT (38%) and liver transplant (50%) recipients with toxoplasmosis were significantly lower than those for the general HSC (84%) and liver transplant (75%) populations (p<0.05) (Table 4).

**Table 5:** Survival among transplant patients with toxoplasmosis, according to patients’ characteristics, Europe, 2010–2014

Characteristic	2-mo survival			6-mo survival	
No. patients/no. survived (%)	p value*	No. patients/no. survived (%)	p value*
All patients	61/87 (70)	Not applicable		46/87 (53)	Not applicable
Chemoprophylaxis					
Yes	30/35 (86)	<0.05		26/35 (74)	<0.01
No	27/43 (63)			16/43 (37)	
Recipient serologic status					
Positive	27/45 (60)	<0.05		15/45 (33)	<0.001
Negative	22/25 (88)			20/25 (80)	
Type of graft					
Hematopoietic stem cell	36/58 (62)	<0.05		22/58 (38)	<0.001
Solid organ	25/29 (86)			24/29 (83)	

*Exact χ^2^ test.

Transplant recipients in whom toxoplasmosis developed were less likely to survive if they were not receiving chemoprophylaxis before or at onset of disease (p<0.05 at 2 months and p<0.01 at 6 months after disease onset) (Table 5); this finding was particularly common among SOT recipients (p<0.05) (Table 3). However, despite chemoprophylaxis, the outcome remained poorer for HSCT patients than for SOT patients (Tables 3, 5).

## Discussion

We provide an overview of practices used to prevent toxoplasmosis in transplant patients in Europe. Despite the well-recognized risk linked to either endogenous reactivation or to transplantation of a cyst-containing organ, prevention policies seem heterogeneous among countries. Serologic screening of solid organ or hematopoietic stem cell donors for *Toxoplasma*, although not mandatory in all countries, seems to be general practice, probably as a result of recommendations of national societies of transplantation, and is mandatory when organs are exchanged between countries. Similarly, pretransplant serologic screening of recipients, although also not mandatory in all countries, was reportedly performed by nearly all responding centers. However, for 17 cases, the serologic status of the recipient or donor was not available in medical charts. Management practices regarding chemoprophylaxis based on donor and recipient serologic results vary substantially, particularly for kidney and liver transplant patients. Indeed, only 35 (50%) of 70 recipients had received chemoprophylaxis, although it was indicated either because of *Toxoplasma* mismatch (SOT) or seropositivity (HSCT). Only 4 (36%) of 11 SOT patients with D+/R– serologic results had received chemoprophylaxis. These 4 patients were all alive 6 months after transplantation. However, our study did not address long-term survival, which at 5 years after transplantation was reportedly poorer for D+/R– than for D–/R– heart transplant recipients (*17*). In that study, Chehrazi-Raffle et al. (*17*) did not record the duration of chemoprophylaxis, a parameter that could be of greater interest. Similarly, only 18 HSCT patients received chemoprophylaxis, although 45 were known to be seropositive (Table 3). Our study also did not address long-term disabilities resulting from toxoplasmosis.

Even with this limited number of cases reported by the participating centers, our study provides some helpful insights and useful data. From a diagnosis point of view, our findings confirm that PCR has become an essential microbiological tool for investigating active infection, as already emphasized in previous studies (*18*,*19*). Indeed, we can confirm that 9 (10.3%) of the 87 cases of toxoplasmosis were diagnosed by PCR in patients with fever only; thus, earlier treatment could be commenced before more serious complications developed; these patients were mostly HSCT recipients. PCRs on blood from 26 patients with no obvious clinical signs were also positive. This finding is consistent with previously reported findings for allo-HSCT patients in centers where routine monitoring by PCR of blood is conducted for several months after transplantation (*14*–*16*,*20*,*21*). Martino et al. (*22*) concluded that clinical toxoplasmosis evolved in about one third of these patients and that early treatment increased survival rates. In our study, survival rates were poor among patients who were asymptomatic at the time of diagnosis (58%) (Table 1), probably because as allo-HSCT patients they were at high risk for death from other causes. Our study did not record what treatment decisions were taken as a direct result of PCR results, and so a more detailed future study of treatment regimens and how quickly they were initiated may provide further valuable insights into factors affecting mortality rates in this clinical group.

Not surprisingly, among the 87 patients, the proportion with disseminated and pulmonary toxoplasmosis was high; this clinical picture is known to be frequent among transplant patients (*4*,*23*,*24*). The high frequency (100%) of positive PCR results among patients with cerebral toxoplasmosis differs from previous estimates of sensitivity in this clinical setting (*2*), suggesting high circulating parasite loads, late diagnoses, or both, which could account for the unusually high mortality rate (85%) among patients with cerebral toxoplasmosis in this study. Another explanation is that diagnostic sensitivity of molecular diagnosis has been mainly evaluated in HIV-infected patients, a patient population that differs from transplant recipients and experiences more severe disease with rapid dissemination of the parasites. On the other hand, ocular toxoplasmosis, a mild form of the disease, occurred mostly after the first year after transplantation, when immune suppressive therapy is usually reduced, thus explaining the 100% survival rate, probably resulting from confinement of parasites in the ocular compartment (*25*).

In HSCT patients, *Toxoplasma* reactivation predominantly occurred within several months (20.6 ± 4.6 weeks) after engraftment, which might suggest that chemoprophylaxis was stopped too early. Indeed, toxoplasmosis was diagnosed for 9 HSCT patients after chemoprophylaxis was stopped (Table 3); this finding is consistent with the policy at 9 centers of discontinuing chemoprophylaxis at 6 months. These data support the practice of monitoring CD4+ T-cell counts to guide chemoprophylaxis discontinuation, as suggested by others (*13*). However, toxoplasmosis was also diagnosed during chemoprophylaxis for 9 additional HSCT and 5 SOT patients, which might be related to inadequate regimens of cotrimoxazole or poor observance. A recent systematic review (*13*) reported that breakthrough toxoplasmosis in HSCT patients was observed when cotrimoxazole was given only 2 times per week at a dosage of 960 mg (57% of cases) or 480 mg daily (18%).

A major finding of this study is the observation that life-threatening toxoplasmosis can occur in HSCT and SOT patients after chemoprophylaxis is stopped. However, in SOT patients, the rather late occurrence after transplant (>3 years) and the high survival rates suggest that infection acquired long after transplantation is usually mild and the source is probably contaminated food. Conversely, life-threatening early infection was associated with a high mortality rate and was mostly observed in liver transplant patients, suggesting that serologic results might not have been taken into account to guide chemoprophylaxis.

Overall, prognosis of *Toxoplasma* infection was good for SOT patients; the all-cause mortality rate of 17% was similar to that reported from Spain (13.6%), where 17 of 22 patients had a primary-acquired infection (*6*). Higher prevalence and severity of disease was confirmed among HSCT patients; survival rate was only 38% at 6 months, similar to mean survival rates recently reported (*13*). We assume that death was attributable to toxoplasmosis in deceased HSCT and liver transplant patients because their 6-month survival rate was significantly poorer than that of their counterparts without toxoplasmosis (p<0.01). A similar effect of toxoplasmosis on survival of HSCT patients has been recently demonstrated in a case–control study (*26*). However, whether chemoprophylaxis positively influences outcome remains unclear. Indeed, overall survival rates were better among patients who received cotrimoxazole than among those who received no treatment; but when considering HSCT and SOT patients separately, survival rates remained significantly better for SOT patients only. This finding raises the question of the effectiveness of prophylaxis, in terms of regimen and duration.

This study has several limitations. First, we used aggregated data, so individual analyses or modifications of the analysis plan were not possible after data collection. Therefore, individual data such as immunosuppressive regimen, graft versus host disease, or simultaneous infections were not recorded, and multivariate analyses to further explore mortality rates were not possible. The number of participating centers per country varied, and for some countries (particularly Germany and Turkey), these centers accounted for a small proportion of the transplantation activity in the whole country (Technical Appendix); thus, we cannot be sure that the data collected were representative for the whole country. The absence of correlation between seroprevalence and the number of cases reported among countries may be attributed to several confounding factors, such as 1) good management of prevention in countries where seroprevalence is high, 2) lack of awareness and possible underdiagnoses of *Toxoplasma*-associated risk in countries where seroprevalence or transplantation activity is low, 3) underreporting because of lack of follow-up, 4) overdiagnosis because of systematic screening (asymptomatic cases), or 5) migration of patients from eastern Europe (higher seroprevalence) to western Europe for transplantation (*27*).

Overall, this study confirms that toxoplasmosis in transplant recipients is a clinical problem throughout Europe, regardless of local seroprevalence. This finding suggests that substantial health gains may be achieved by the development and adoption of common prevention guidelines based on best practice. Whether chemoprophylaxis duration should be extended and for what duration remains to be determined. Nevertheless, our results suggest that to prevent late onset of toxoplasmosis, cotrimoxazole should be given for >6 months. In case of drug intolerance, low dosage, or discontinuation, follow-up by regular PCR of blood could help guide preemptive treatment. In SOT patients with *Toxoplasma* mismatch (D+/R–), cotrimoxazole prophylaxis should be given for >1 year. Last, recommendations associated with hygiene, similar to those provided to seronegative pregnant women to avoid contamination, should be extended to all seronegative transplant patients.

Technical Appendix Characteristics of hematopoietic stem cell and solid organ transplant cases, Europe, 2010–2014.
