# Supplementary material for: Toxoplasmosis in Transplant Recipients, Europe, 2010–2014
Source: Emerg Infect Dis. 2018 Aug;24(8):1497–504. doi: 10.3201/eid2408.180045 (PMC6056100; doi:10.3201/eid2408.180045)
Supplement: Technical Appendix — Characteristics of hematopoietic stem cell and solid organ transplant cases, Europe, 2010–2014. [file 18-0045-Techapp-s1.pdf]

Article DOI: https://doi.org/10.3201/eid2408.180045

Toxoplasmosis in Transplant Recipients, Europe, 2010–2014

Technical Appendix

Technical Appendix Table. Characteristics among responding countries

	Country										
Characteristic	France	Spain	Italy	Switzerland	Romania	Serbia	Germany	Slovaquia	Greece	Turkey	United Kingdom
No of participating centers	6	8	10	6	2	2	1	3	1	2	5
Annual No of grafts[a], responding centers/whole country (%)											
Allo-HSC	320/1900 (17)	60/900 (6)	136/1630 (8)	226 (100)	35/35 (100)	13/13 (100)	155/3141 (5)	50/50 (100)	Un	40/1180 (4)	250/1678 (15)
Liver	346/1241 (28)	307/1100 (28)	182/1000 (18)	40/100 (40)	120/120 (100)	Na	Un	Na	35/35 (100)	113/670 (17)	272/710 (38)
Kidney	635/3232 (20)	663/2550 (26)	157/1600 (10)	80/280 (29)	130/310 (42)	30/75 (40)	Un	80/150 (53)	40/55 (73)	141/1702 (8)	560/3064 (18)
Heart	93/420 (22)	105/250 (42)	81/230 (35)	12/35 (33)	Un	5/10 (50)	Un	10/18 (56)	0/9 (0)	6/85 (7)	82/190 (41)
No of reported cases	58	4	8	3	1	6	3	4	0	0	0
No of asymptomatic cases	23	0	3	1	0	4	0	0	Na	Na	Na
No of cases/1000 grafts [b]											
Allo-HSC	26.2	0	11.2	0	Un	92.3	3.9	Un	Un	Na	Na
Liver	2.9	1.3	Un	4	Un	Un	Un	Un	0	Na	Na
Kidney	2.2	0	9	0	1.5	0	Un	Un	0	Na	Na
Heart	8.6	8.3	3.2	5.7	Un	0	Un	80	Un	Na	Na
Cotrimoxazole preferred regimen											
Allo-HSC	960mgx3/wk	960mgx3/wk[c]	960mgx3/wk	960mgx3/wk	960mgx3/wk	480mgx3/wk	480mgx2/wk	960mgx3/wk	Un	960mgx3/wk[c]	960mgx3/wk
Liver	960mgx3/wk	480 mg/d	480 mg/d	960mgx3/wk	Na	Na	Un	Na	480 mg/d	960mgx3/wk[d]	480mgx3/wk
Kidney	480 mg/d	480 mg/d	480 mg/d	960mgx3/wk[d]	Na	960 mg/d	Un	480 mg/d	480mgx3/wk	480 mg/d	480mgx3/wk
Heart	960mgx3/wk	480 mg/d	960mgx3/wk	960mgx3/wk	Un	960mgx3/wk	Un	480mgx3/wk	480mgx3/wk	480 mg/d	960mgx3/wk
Seroprevalence, %	50	30	50	35	60	31	50	50	30	47	20
Pretransplant serology [e]											

	Country										
Characteristic	France	Spain	Italy	Switzerland	Romania	Serbia	Germany	Slovaquia	Greece	Turkey	United Kingdom
Allo-HSC	5/5	3/3	5/5	3/3	1/1	1/1	1/1	0/2	Un	1/1	4/4
Liver	5/5	7/7	3/5	3/3	1/1	Na	Un	Na	1/1	2/2	2/2
Kidney	6/6	7/7	4/6	6/6	1/1	1/1	Un	1/2	1/1	2/2	2/3
Heart	5/5	6/6	4/4	3/3	Na	1/1	Un	1/1	1/1	1/1	3/4

[a] mean transplantation activity over the study period (2010–2014); [b] number of cases divided by the number of grafts in the centers that participated to case report, cumulative data over the study period (2010–2014); [c] minimal doses or intakes; [d] or 480 mg/d indifferently; [e] No of centers with screening/no of centers dealing with the type of transplant

Un, unknown data (no participating center or no participation in case report); Na, not applicable (no such transplantation in the country); wk, week; d, day.
